# *Didelphis* spp. opossums and their parasites in the Americas: A One Health perspective

**DOI:** 10.1007/s00436-021-07072-4

**Published:** 2021-03-31

**Authors:** Marcos Antônio Bezerra-Santos, Rafael Antonio Nascimento Ramos, Artur Kanadani Campos, Filipe Dantas-Torres, Domenico Otranto

**Affiliations:** 1 Department of Veterinary Medicine, University of Bari, Valenzano, 70010 Bari, Italy; 2 Laboratory of Parasitology, Federal University of the Agreste of Pernambuco, Garanhuns, Brazil; 3 Department of Veterinary Medicine, Federal University of Viçosa, Viçosa, Brazil; 4 Aggeu Magalhães Institute, Fundação Oswaldo Cruz (Fiocruz), Recife, Brazil; 5 Department of Pathobiology, Faculty of Veterinary Science, Bu-Ali Sina University, Hamedan, Iran

**Keywords:** *Didelphis* spp., Public health, Reservoirs, Vectors, Wildlife, Zoonotic parasites

## Abstract

Medium sized opossums (*Didelphis* spp.) are among the most fascinating mammals of the Americas, playing important ecological roles (e.g., dispersal of seeds and control of insect populations) in the environment they inhabit. Nevertheless, as synanthropic animals, they are well adapted to human dwellings, occupying shelters within the cities, peripheral areas, and rural settings. These marsupials can harbor numerous pathogens, which may affect people, pets, and livestock. Among those, some protozoa (e.g., *Leishmania infantum*, *Trypanosoma cruzi*, *Toxoplasma gondii*), helminths (e.g., *Ancylostoma caninum*, *Trichinella spiralis*, *Alaria marcianae*, *Paragonimus* spp.) and arthropods (e.g., ticks, fleas) present substantial public health and veterinary importance, due to their capacity to cause disease in humans, domestic animals, and wildlife. Here, we reviewed the role played by opossums on the spreading of zoonotic parasites, vectors, and vector-borne pathogens, highlighting the risks of pathogens transmission due to the direct and indirect interaction of humans and domestic animals with *Didelphis* spp. in the Americas.

## Introduction

**Fig. 1:**
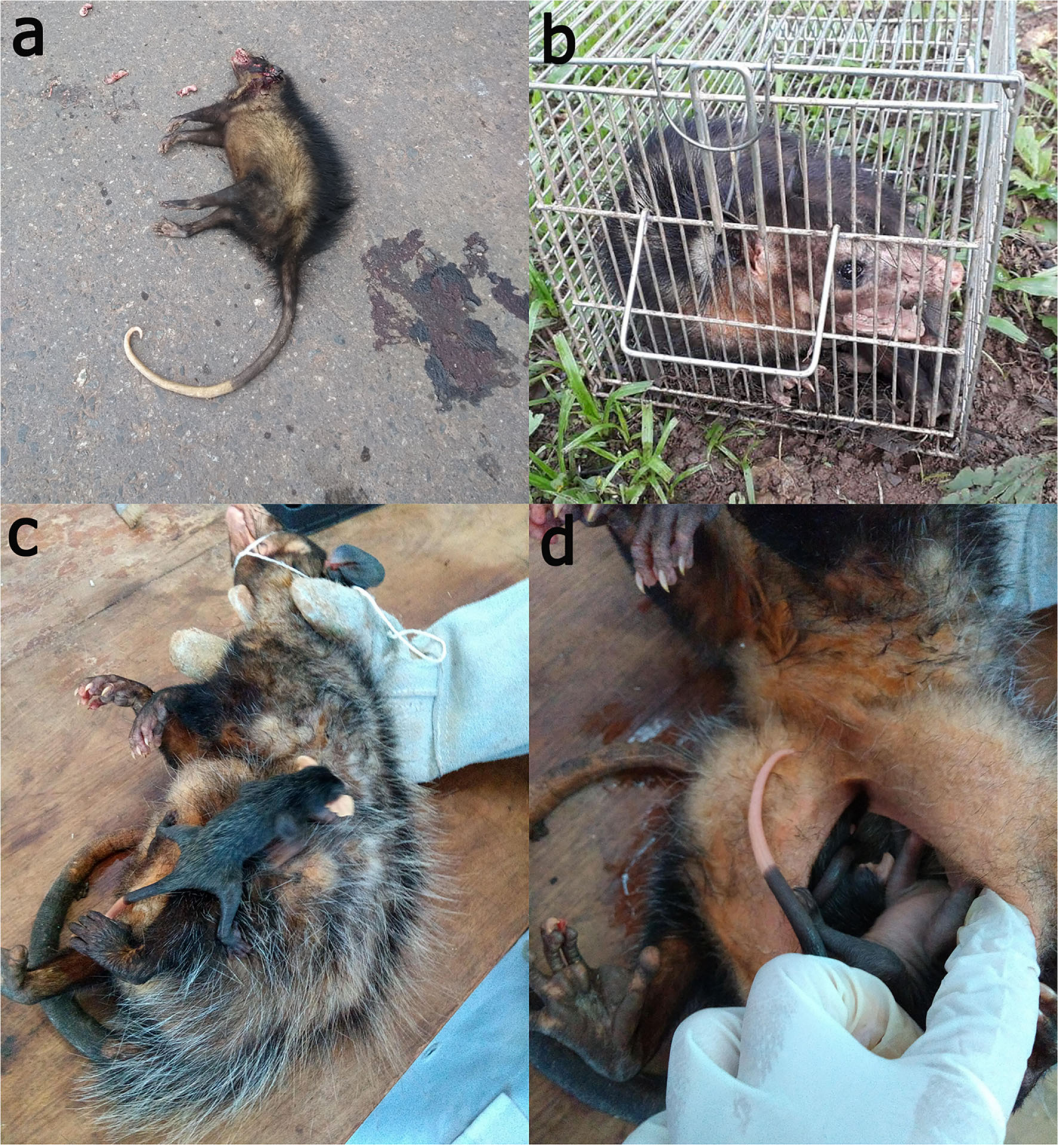
**a**. Road killed *Didelphis aurita* female with its marsupium full of joeys; **b**. *Didelphis aurita* trapped through Tomahawk livetrap; **c**. *Didelphis aurita* female with joeys on its back; **d**. *Didelphis aurita* female with joeys inside the marsupium.

Over history, uncontrolled environmental changes promoted by humans have led to irreversible outcomes, affecting natural resources and consequently the biotic populations of the modified landscape (Acevedo-Whitehouse and Duffus 2009; Dantas-Torres 2015). For example, the impact of deforestation to give place to productive activities (e.g., livestock system and cropping) and construction of large cities has resulted in the extinction of wildlife species due to habitat loss and fragmentation of their populations (Rands et al. 2010; Pereira et al. 2010; Haddad et al. 2015). However, some species, such as medium sized opossums of the genus *Didelphis* (from this point will be referred in the text only as opossums), can adapt to human-modified landscapes due to their ability to exploit a wide range of resources and environments (Cruz-Salazar and Ruiz-Montoya 2020). The presence of such animals within human dwellings across the American continent brings important consequences (i.e., disease transmission) not only for humans and domestic animals, but also for themselves as they are victims of domestic animal attacks, roadkill (Fig. 1a) or directly killed by humans (Gumier-Costa and Sperber 2009; Rangel and Neiva 2013; Barros and Azevedo 2014).

Opossums’ ability to thrive in different environments enabled them to become synanthropic species, benefiting from resources (e.g., food and shelter) available in human-modified areas (Olifiers et al. 2005; Jansen and Roque 2010). These marsupials are compatible hosts and suggested reservoirs of important parasites (e.g., *Leishmania infantum* and *Trypanosoma cruzi*) that cause human disease, and along with domestic animals, opossums are involved in the epidemiological cycle of such parasites within urban and periurban environments (Muller et al. 2005; Horta et al. 2010; Pena et al. 2011; Carreira et al. 2012). Additionally, opossums are also potential amplifiers of some pathogens, such as *Rickettsia rickettsii* (Horta et al. 2009).

Several pathogens present in wildlife are still unknown to science, and in many countries the illegal consumption of these animals exposes humans to infectious agents harbored by wildlife (Chomel et al. 2007; Júnior et al. 2010; Bezerra-Santos et al. 2021a, b). For example, opossums have been consumed by local communities as food or even as traditional medicine (e.g., opossum fat) in some regions of the Americas, which in many situations lead to local illegal trade of these animals (Júnior et al. 2010; Barros and Azevedo 2014; de Oliveira Carneiro et al. 2019). Such habits impose substantial health risks for people consuming or handling opossums’ meat/carcasses, due to lack of monitoring of the health status of these animals, poor hygienic conditions when manipulating their meat (e.g., cross contamination due to the contact of the animals’ feces, blood, saliva, urine with other food items such as raw vegetables) and inadequate cooking. Here, we reviewed the role played by opossums on the spreading of zoonotic parasites, vectors and vector-borne pathogens, highlighting the veterinary and public health consequences that the direct and/or indirect contact with these animals may bring for people and domestic animals.

## The genus *Didelphis*: ecology and coexistence with domestic animals and humans

**Fig. 2:**
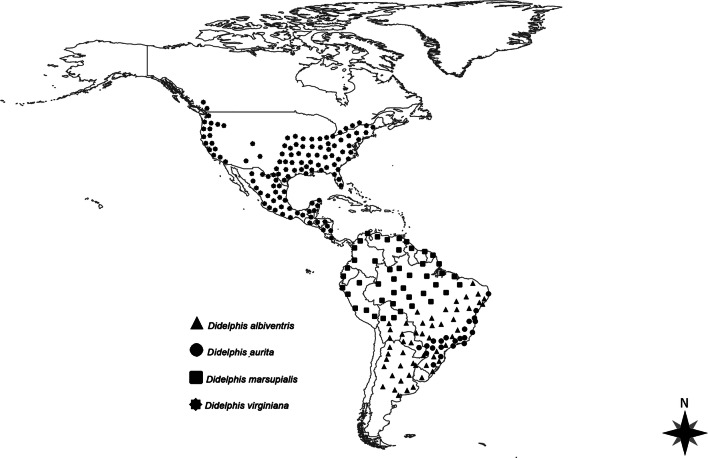
Geographical distribution of the four major *Didelphis* spp. in the Americas (Gardner 2008; Pérez-Hernandez et al. 2016).

The genus *Didelphis* consist of six species generally known as “New World marsupials” or “opossums” (Voss and Jansa 2009). Except for *Didelphis virginiana,* these animals are classified in two groups: *Didelphis marsupialis*-group (i.e., *Didelphis marsupialis* and *Didelphis aurita*) and *Didelphis albiventris*-group (i.e., *Didelphis albiventris*, *Didelphis pernigra* and *Didelphis imperfecta*) (Gardner 2008; Faria and de Melo 2017). From the species up to date recognized, *D. albiventris*, *D. aurita*, *D. marsupialis* and *D. virginiana* are the most abundant and widely distributed (Fig. 2). Three of them are mostly restricted to South America: the white-eared opossum (*D. albiventris*) occurring in Argentina, Bolivia, Brazil, Paraguay, and Uruguay; the black-eared opossum (*D. aurita*), found in northeastern Argentina, eastern Brazil, and southeastern Paraguay; and the common opossum (*D. marsupialis*), which is distributed through Trinidad and Tobago, the Guianas, Mexico, and in the Amazon basin (including Bolivia, Brazil, Colombia, Ecuador, Peru, and Venezuela). Conversely, the Virginia opossum (*D. virginiana*) is widely distributed and abundant in North and Central America, being reported from Canada to Costa Rica (Gardner 2008). The other two species (i.e., *D. imperfecta* and *D. pernigra*) have a more limited distribution being the first restricted to small land range in Venezuela, Brazil, Surinam, and the Guianas, and the second found throughout the Andes region (Gardner 2008).

Species of the genus *Didelphis* have twilight and nocturnal habits and are considered synanthropic animals due to the high adaptation to human dwellings, being frequently found on the roof of houses, hollows of trees and other shelters within the cities and peripheral areas (Jansen 2002). Due to their circulation in urban and rural environments, opossums are considered potential reservoirs of many infectious agents (e.g., *Trypanossoma cruzi*, *Leishmania infantum*, *Rickettsia* spp., *Ancylostoma caninum*, *Angiostrongylus cantonensis*, *Angiostrongylus costaricensis*) to humans and domestic animals (Miller et al. 2006; Horta et al. 2009, 2010; Carreira et al. 2012; Cantillo-Barraza et al. 2015; Dalton et al. 2017; Bezerra-Santos et al. 2020a). Additionally, ectoparasites (e.g., ticks and fleas) harbored by these animals have been reported as vectors of important arthropod-borne zoonotic pathogens (e.g., *Rickettsia* spp. and *Borrelia* spp.) (Hanincová et al. 2006; Horta et al. 2009; Abramowicz et al. 2012; Maina et al. 2016).

Opossums present omnivorous habit and opportunistic feeding behavior, with a diet consisting of small vertebrates, invertebrates, seeds, and fruits (Cáceres and Monteiro-Filho 2001). The feeding behavior of these animals varies according to the seasons of the year, depending on the availability of resource found in each period. For instance, during the rainy season they have higher food intake of fruits and vertebrate animals. Additionally, the diet of these marsupials may differ according to the environment where they are mostly inserted, being possible to record fruits and garbage remnants of human consumption, as well as food only available inside forest fragments (Cáceres and Monteiro-Filho 2001). This opportunistic feeding behavior exposes them to diverse parasitic infections (Jiménez et al. 2011). For instance, by consuming contaminated food from garbage remnants (e.g., garbage contaminated with feces of infected dogs or cats) they may get gastrointestinal parasite infections (e.g., *Ancylostoma caninum* and *Toxocara* spp.), and the ingestion of insect vectors (e.g., kissing bugs) may expose them to blood protozoa such as *Trypanosoma cruzi* (Schweigmann et al. 1995; Teodoro et al. 2019; Bezerra-Santos et al. 2020a).

These marsupials live in close relationship with humans and domestic animals. In many South American countries, some people consider opossums as pests, frequently mistaken them as rats or by considering them dirty animals (Krause and Krause 2006; Barros and Azevedo 2014). This factor leads to their death within the cities using poison or even traps to capture and kill (Fig. 1b) (Stone et al. 2000; Barros and Azevedo 2014). Domestic animals (predominantly dogs) also use to attack opossums, especially during the reproduction period, in which females are slower due to the high number of baby opossums (known as “joeys”) they carry on their back or within the marsupium (Fig. 1c, d) (Rangel and Neiva 2013). In rural settings, mainly in poultry farms, opossums are also seen as a problem, since they have been considered important predators of domestic birds, leading to human and wildlife conflicts (Amador-Alcalá et al. 2013).

In their natural habitats, opossums have an irreplaceable ecological role acting in the control of pests (e.g., insects and venomous animals), as well as in the dispersion of seeds, which contribute to the ecology of various plant species because of their mutualistic interactions (Cantor et al. 2010; Voss and Jansa 2012). This behavior makes opossums important for the natural reforestation process of disturbed forest fragments (Cantor et al. 2010). As such, regardless their role in the ecology of zoonotic parasites, these marsupials are not human’s enemies, as they are essential contributors for the ecosystem in which they are included. However, given their reservoir role of infectious agents, it is extremely important the establishment of human awareness regarding the prevention of diseases caused by pathogens transmitted by opossums.

## Arthropods and vector-borne pathogens associated with *Didelphis* spp.

### Ticks and tick-borne pathogens

Ticks are among the vectors of major importance in the transmission of pathogens (e.g., *Babesia* spp., *Ehrlichia* spp., *Anaplasma* spp., *Rickettsia* spp. and *Borrelia* spp.) (Dantas-Torres et al. 2012a; Otranto 2018). These blood-sucking arthropods feed on a great variety of vertebrate hosts, being recorded in humans, domestic animals, and wildlife, including opossums (Soares et al. 2015; Saracho-Bottero et al. 2018; Mendoza-Roldan et al. 2020a). It is known that the exchange of ectoparasites among these hosts has a central importance in the epidemiology of tick-borne diseases. In this perspective, opossums are suggested to play a central role in the epidemiological chain of some tick-borne pathogens, as one of their behaviors (i.e., movement among forests, urban, and rural locations) facilitates the spreading of different tick species from wild environments to urban sites (Bermúdez et al. 2016; Rojero-Vázquez et al. 2017). For example, opossums captured close to human dwellings have been reported harboring species of hard ticks (*Amblyomma* spp.) (Massini et al. 2019; Bezerra-Santos et al. 2020b), which are usually found in other wildlife species within forests areas of Brazil (Dantas-Torres et al. 2010). Moreover, the relevance of opossums as potential disseminators of tick-borne pathogens has been suggested by studies on the exposure of these marsupials to tick-borne zoonotic pathogens, such as *R. rickettsii*, *B. burgdorferi* and *Ehrlichia chaffeensis* (Horta et al. 2009; Castellaw et al. 2011; Melo et al. 2016). For instance, an experimental study demonstrated that *D. aurita* opossums get infected after being exposed to *R. rickettsii*-positive *Amblyomma sculptum* ticks, developing rickettsemia capable of causing infection in guinea pigs and ticks, and acting as amplifier hosts of this bacterium to ticks, although the infection rate (i.e., 5% to 18%) in ticks was considered low (Horta et al. 2009). On the other hand, despite seroprevalence of 27.5% for *B. burgdorferi* and 15.8% for *E. chaffeensis* have been demonstrated in opossums, the role of these animals in the epidemiology of these bacteria is unclear (Castellaw et al. 2011; Melo et al. 2016).

**Fig. 3:**
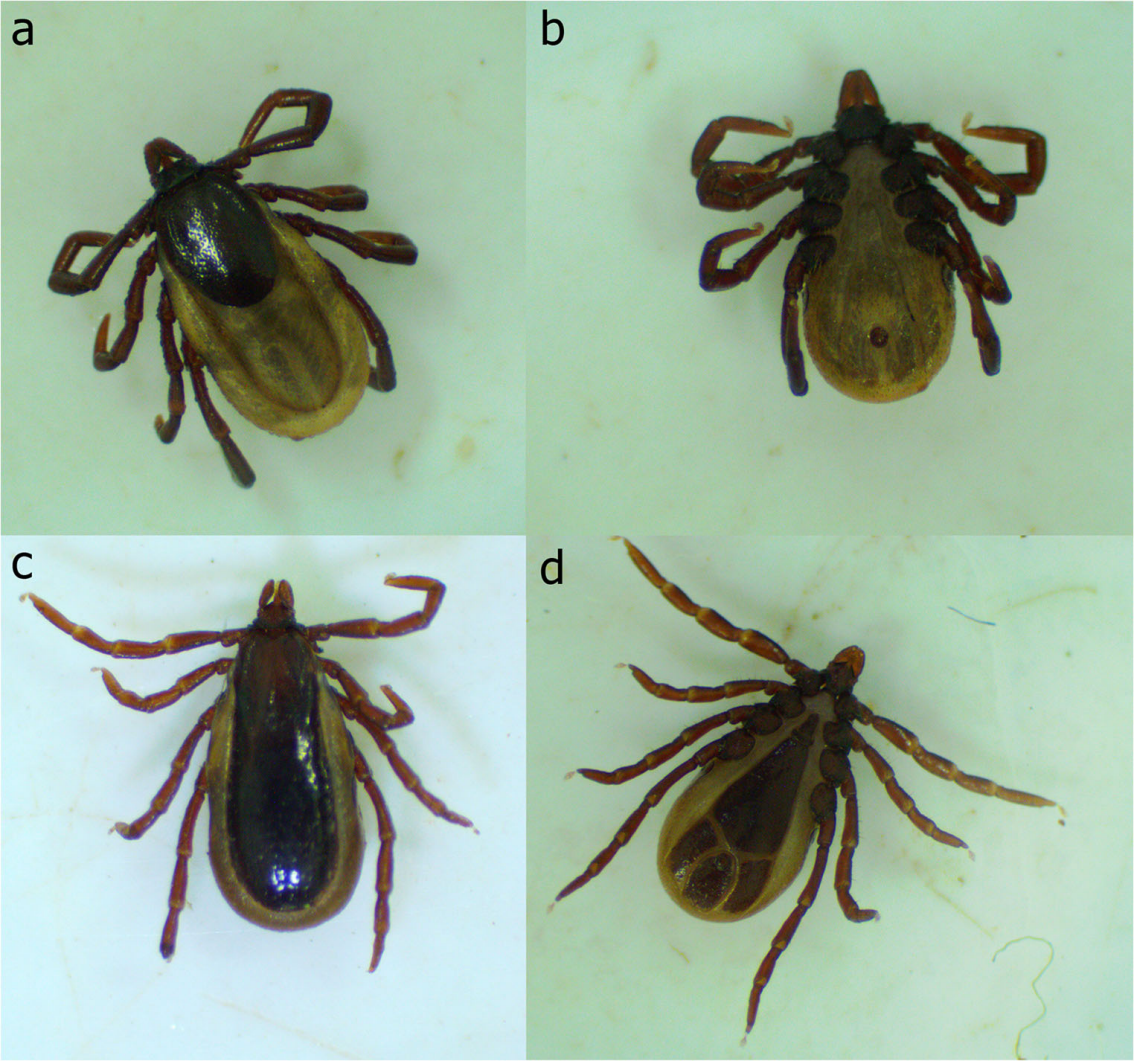
*Ixodes loricatus* adults collected on *Didelphis aurita* opossums. Female dorsal (**a**) and ventral view (**b**). Male dorsal (**c**) and ventral view (**d**).

Despite the genus *Amblyomma* being the most diverse among the ixodid ticks reported on *Didelphis* spp. in South America (Table 1), most life stages detected are larvae and nymphs (Dantas-Torres et al. 2012b; Saraiva et al. 2012; Sponchiado et al. 2015; Lopes et al. 2018). In contrast, the genus *Ixodes* is mostly reported on these marsupials as adult stages (Fig. 3), with the species *Ixodes loricatus*, *Ixodes amarali*, *Ixodes luciae* and *Ixodes didelphidis* having these animals as primary hosts (Labruna et al. 2004, 2009; Dantas-Torres et al. 2012b). Indeed, it is known that they are three host ticks feeding on small rodents at early life stages, and on opossums at the adult stage (Labruna et al. 2009; Nava et al. 2017; Tarragona et al. 2018). Up to date, there is no record on the vector role of these species or their parasitic association with humans. However, transovarial and transstadial transmission of *Rickettsia belli*, a microorganism of unknown pathogenicity, have been demonstrated in engorged ticks, *I. loricatus*, collected from *D. aurita*, suggesting that this tick species could have a role in the maintenance of this bacterium in nature (Horta et al. 2006). Further studies are advocated on the vector role and host range of these species, as some of them (e.g., *I. loricatus*) have been the main and most abundant ticks found on *Didelphis* spp. from urban and rural environments (Dantas-Torres et al. 2012b; Tarragona et al. 2018; Bezerra-Santos et al. 2020b). Thus, identifying their potential role on the epidemiology of tick-borne pathogens affecting humans and/or domestic animals is important from a One Health perspective. A single species of the genus *Haemaphysalis* (i.e., *Haemaphysalis juxtakochi*) has been recorded on *D. aurita* and *D. marsupialis* (Lamattina et al. 2018a, b; Domínguez et al. 2019). This species is known as the neotropical deer tick and has also been reported on other wildlife species, humans, and domestic animals (Costa et al. 2017; Saracho-Bottero et al. 2018). In addition, some zoonotic pathogens such as *Rickettsia parkeri* and *B. burgdorferi* sensu lato have been detected in *H. juxtakochi*, although its role in the transmission of these pathogens to humans is probable negligible (Souza et al. 2018; Flores et al. 2018).

**Table 1:** Ticks reported on *Didelphis* species, occurrence on humans and associated zoonotic pathogens

Marsupial species	Tick species	Occurrence on humans	Associated zoonotic pathogens	Reference
***Didelphis albiventris***	**Ixodidae**			
	*Amblyomma aureolatum*	Yes	Spotted Fever Group Rickettsiae	Muller et al. (2005); Barbieri et al. (2015); Moraes-Filho et al. (2018); Reck et al. (2018)
	*Amblyomma auricularium*	Yes	No record	Fontalvo et al. (2017); Szabó et al. (2020)
	*Amblyomma coelebs*	Yes	No record	Garcia et al. (2015); Sponchiado et al. (2015); De Sá et al. (2018)
	*Amblyomma dubitatum*	Yes	Spotted Fever Group Rickettsiae	Sakai et al. (2014); Matias et al. (2015); Sponchiado et al. (2015); Lamattina and Nava (2016); Reck et al. (2018); De Sá et al. (2018)
	*Amblyomma fuscum*	Yes	No record	Marques et al. (2006); Martins et al. (2009); Dantas-Torres et al. (2012b)
	*Amblyomma ovale*	Yes	Spotted Fever Group Rickettsiae	Barbieri et al. (2015); Sponchiado et al. (2015); Reck et al. (2018); Lamattina et al. (2018a)
	*Amblyomma sculptum*	Yes	Spotted Fever Group Rickettsiae	Sponchiado et al. (2015); Bitencourth et al. (2017); Polo et al. (2017); De Sá et al. (2018); Saracho-Bottero et al. (2018)
	*Amblyomma parkeri*	Yes	*Rickettsia parkeri*	Sponchiado et al. (2015); Zeringóta et al. (2017); Reck et al. (2018); Borsoi et al. (2019)
	*Ixodes loricatus*	No record	No record	Muller et al. (2005); Horta et al. (2007); Dantas-Torres et al. (2012b); da Silva et al. (2017); De Sá et al. (2018)
	**Argasidae**			
	*Ornithodoros mimon*	Yes	No record	Labruna et al. (2014); Sponchiado et al. (2015); da Silva et al. (2017)
***Didelphis aurita***	**Ixodidae**			
	*Amblyomma aureolatum*	Yes	Spotted Fever Group Rickettsiae	Salvador et al. (2007); Barbieri et al. (2015); Luz et al. (2018); Moraes-Filho et al. (2018); Reck et al. (2018)
	*Amblyomma brasiliense*	Yes	No record	Szabó et al. (2006); Szabó et al. (2013); Lamattina et al. (2018a, 2018b)
	*Amblyomma coelebs*	Yes	No record	Saraiva et al. (2012); Garcia et al. (2015); Lamattina et al. (2018a, 2018b)
	*Amblyomma dubitatum*	Yes	Spotted Fever Group Rickettsiae	Horta et al. (2007); Sakai et al. (2014); Matias et al. (2015); Lamattina and Nava (2016); Reck et al. (2018)
	*Amblyomma fuscum*	Yes	No record	Marques et al. (2006); Martins et al. (2009); Dantas-Torres et al. (2012b); Szabó et al. (2013)
	*Amblyomma geayi*	No record	*Rickettsia amblyommatis*	Oliveira et al. 2014; Dolz et al. 2019
	*Amblyomma incisum*	Yes	No record	Szabó et al. (2006); Reck et al. (2018); Lamattina et al. (2018a, b)
	*Amblyomma ovale*	Yes	Spotted Fever Group Rickettsiae	Szabó et al. (2013); Barbieri et al. (2015); Lamattina et al. (2018a, b); Reck et al. (2018)
	*Amblyomma sculptum*	Yes	Spotted Fever Group Rickettsiae	Salvador et al. (2007); Saraiva et al. (2012); Szabó et al. (2013); Bitencourth et al. (2017); Polo et al. (2017); Saracho-Bottero et al. (2018)
	*Amblyomma scutatum*	No record	No record	Oliveira et al. (2014)
	*Amblyomma yucumense*	No record	No record	Krawczak et al. (2015)
	*Haemaphysalis juxtakochi*	Yes	*Rickettsia parkeri, Borrelia burgdorferi* sensu lato	Lamattina et al. (2018a); Reck et al. (2018); Saracho-Bottero et al. (2018); Souza et al. (2018); Flores et al. (2018)
	*Ixodes amarali*	No record	No record	Oliveira et al. (2014)
	*Ixodes auritulus*	No record	*Borrelia burgdorferi* sensu stricto, *Borrelia burgdorferi* sensu lato	Morshed et al. (2005); Oliveira et al. (2014); Carvalho et al. (2020)
	*Ixodes didelphidis*	No record	No record	Barros-Battesti et al. (2000); Oliveira et al. (2014)
	*Ixodes loricatus*	No record	No record	Horta et al. (2007); Salvador et al. (2007); Dantas-Torres et al. (2012b); Saraiva et al. (2012); Szabó et al. (2013); Oliveira et al. (2014); Luz et al. (2018)
	*Ixodes luciae*	No record	No record	Oliveira et al. (2014)
***Didelphis marsupialis***	**Ixodidae**			
	*Amblyomma cajennense* sensu stricto	No record	*Rickettsia amblyommatis*	Costa et al. (2017); Binetruy et al. (2019)
	*Amblyomma coelebs*	Yes	No record	Garcia et al. (2015); Witter et al. (2016); Binetruy et al. (2019)
	*Amblyomma dissimile*	Yes	*Rickettsia monacensis*	Guglielmone et al. (2006); Domínguez et al. (2019); Mendoza-Roldan et al. (2021)
	*Amblyomma geayi*	No record	*Rickettsia amblyommatis*	Soares et al. (2015); Dolz et al. (2019)
	*Amblyomma humerale*	No record	*Rickettsia amblyommatis*	Soares et al. (2015); Witter et al. 2016, Binetruy et al. (2019); Gruhn et al. (2019)
	*Amblyomma mixtum*	Yes	*Rickettsia rickettsii, Coxiella Burnetii*	Bermúdez et al. (2016); Noda et al. (2016); Rodríguez-Vivas et al. (2016); Domínguez et al. (2019)
	*Amblyomma oblongoguttatum*	Yes	*Rickettsia amblyommatis*	Bermúdez et al. (2012); Aguirre et al. (2018); Domínguez et al. (2019)
	*Amblyomma ovale*	Yes	Spotted Fever Group Rickettsiae	Barbieri et al. (2015); Lamattina et al. (2018b); Reck et al. (2018); Domínguez et al. (2019)
	*Amblyomma pacae*	Yes	No record	Guglielmone et al. (2006); Soares et al. (2015)
	*Amblyomma parkeri*	Yes	*Rickettsia parkeri*	Witter et al. (2016); Zeringóta et al. (2017); Reck et al. (2018); Borsoi et al. (2019)
	*Amblyomma sabanerae*	Yes	No record	Bermúdez et al. (2012); Domínguez et al. (2019)
	*Amblyomma sculptum*	Yes	Spotted Fever Group Rickettsiae	de Lemos et al. (1996); Bitencourth et al. (2017); Polo et al. (2017); Saracho-Bottero et al. (2018)
	*Amblyomma triste*	Yes	*Rickettsia parkeri*	de Lemos et al. (1996); Guglielmone et al. (2006); Melo et al. (2015)
	*Haemaphysalis juxtakochi*	Yes	*Rickettsia parkeri; Borrelia burgdorferi* sensu lato	Flores et al. (2018); Souza et al. (2018); Reck et al. (2018); Saracho-Bottero et al. (2018); Domínguez et al. (2019)
	*Ixodes didelphidis*	No record	No record	Abel et al. (2000)
	*Ixodes loricatus*	No record	No record	Abel et al. (2000)
	*Ixodes luciae*	No record	No record	Domínguez et al. (2019); Binetruy et al. (2019)
	*Rhipicephalus sanguineus* sensu lato	Yes	*Rickettsia rickettsii*, *Bartonella henselae, Rickettsia conorii*	Goddard (1989); Kollars (1993); Matsumoto et al. (2005); Dantas-Torres et al. (2006); Wikswo et al. (2007); Otranto et al. (2014); Bermúdez et al. (2016); Reck et al. (2018)
***Didelphis virginiana***	**Ixodidae**			
	*Amblyomma americanum*	Yes	*Ehrlichia chaffeensis*, *Ehrlichia ewingii*, *Rickettsia rickettsii*, *Coxiella burnetii*, *Francisella tularensis*, *Borrelia lonestari*, *Rickettsia parkeri*	Kollars (1993); Lavender and Oliver (1996); Oliver et al. (1999); Childs and Paddock (2003); Levin et al. (2017)
	*Dermacentor variabilis*	Yes	Spotted Fever Group Rickettsiae, *Francisella tularensis, Ehrlichia chaffeensis*, *Ehrlichia ewingii*	Fish and Dowler (1989); Kollars (1993); Lavender and Oliver (1996); Oliver et al. (1999); Steiert and Gilfoy (2002); Stromdahl et al. (2011); Hecht et al. (2019); Whitten et al. (2019)
	*Ixodes affinis*	No record	*Borrelia burgdorferi, Borrelia bissettiae, Bartonella henselae*	Lavender and Oliver (1996); Maggi et al. (2019)
	*Ixodes cookei*	Yes	*Borrelia burgdorferi, Babesia microti*	Fish and Dowler (1989); Hall et al. (1991); Scott et al. (2019)
	*Ixodes scapularis*	Yes	*Borrelia burgdorferi* sensu stricto, *Borrelia mayoni, Borrelia miyamoto*, *Ehrlichia muris eauclarensis*, *Babesia microti*	Kollars (1993); Lavender and Oliver (1996); Oliver et al. (1999); Eisen and Eisen (2018); Xu et al. 2019
	*Ixodes texanus*	Yes	*Borrelia burgdorferi, Rickettsia rickettsii*	Anderson et al. (1986); Fish and Dowler (1989); Hall et al. (1991); Ouellette et al. (1997)
	*Haemaphysalis longicornis*	Yes	severe fever with thrombocytopenia syndrome virus*, Anaplasma* spp.*, Rickettsia* spp*., Babesia* spp.*, Theileria* spp*., Borrelia* spp*.*	Tufts et al. (2020)
	*Rhipicephalus sanguineus* sensu lato	Yes	*Rickettsia rickettsii*, *Bartonella henselae, Rickettsia conorii*	Goddard (1989); Kollars (1993); Matsumoto et al. (2005); Dantas-Torres et al. (2006); Wikswo et al. (2007); Otranto et al. (2014); Reck et al. (2018)
***Didelphis imperfecta***	**Ixodidae**			
	*Amblyomma cajennense* sensu stricto	No record	*Rickettsia amblyommatis*	Costa et al. (2017); Binetruy et al. (2019)

Different tick species have been reported on the Virginia opossum due to its distinct geographical range as compared to other species of the same genus (Fig. 2). Up to date, *Amblyomma americanum*, *Dermacentor variabilis*, *Haemaphysalis longicornis*, and five species of the genus *Ixodes* have been recorded on this opossum (Table 1). However, despite the medical and veterinary importance of these ticks, their relationship with opossums needs further investigation, particularly regarding their ability to acquire pathogens from opossums, as well as the capacity of opossums to serve as amplifier hosts. Most recently, the Virginia opossum has been found commonly infested by *H. longicornis* (Table 1), an invasive Asian tick, in some states of the USA (Tufts et al. 2020; White et al. 2020). This tick species has been associated with several vector-borne pathogens (e.g., *Anaplasma* spp., *Rickettsia* spp., *Babesia* spp., *Theileria* spp., *Borrelia* spp.) in its native range. However, its potential to serve as vector for pathogens of medical and veterinary importance in USA is still poorly understood (Tufts et al. 2020). Indeed, under laboratory conditions, *H. longicornis* larvae and nymphs became infected with *R. rickettsii* after feeding on infected guinea pigs and were able to transmit them to naïve guinea pigs (Stanley et al. 2020). In addition, the same study also demonstrated the transstadial and transovarial transmission of *R. rickettsii* in the same tick species (Stanley et al. 2020). Lastly, *Rhipicephalus sanguineus* sensu lato (s.l.) infesting opossums are rare, being described only in *D. virginiana* and *D. marsupialis* (Kollars 1993; Bermúdez et al. 2016). Thus, the capacity of opossums to maintain this tick species population, as well as the sharing of pathogens have not been assessed and seem unlikely.

### Fleas and flea-borne pathogens

Opossums harbor a great diversity of flea species (Horta et al. 2007; Pinto et al. 2009; Urdapilleta et al. 2019; Bezerra-Santos et al. 2020b; Canto-Osorio et al. 2020), with some of them being regarded as important vectors of zoonotic pathogens (e.g., *Ctenocephalides felis felis*, *Xenopsylla cheopis*, *Pulex irritans* and *Pulex simulans*), while others (e.g., *Adoratopsylla antiquorum*, *Polygenis occidentalis* and *Cediopsylla simplex*) presenting unknown vector role as studies on their ecology and epidemiology are scanty (Table 2). Among the pathogens transmitted by fleas reported on *Didelphis* spp., *R. typhi, R. felis* and *Y. pestis* are of major public health concern (Azad et al. 1997; Demeure et al. 2019; Oliveira et al. 2020). *Rickettsia typhi* is the etiological agent of the murine typhus, a worldwide distributed zoonosis with a life cycle involving rodents (*Rattus rattus* and *Rattus norvegicus*) as vertebrate hosts, and fleas (*X. cheopis*) as vectors (Peniche-Lara et al. 2015). This pathogen has also been reported through molecular methods in *C. felis felis*, *Leptopsylla segnis*, *Ctenophthalmus congeneroides* and *Rhadinopsylla insolita* (Peniche-Lara et al. 2015). The transmission occurs from rodents to humans through contaminated feces of infected fleas. Despite the rodents being the main hosts of *R. typhi*, opossums were infected with this bacterium in the USA and are believed to have important role on the epidemiology of the disease (Williams et al. 1992; Sorvillo et al. 1993; Brown and Macaluso 2016). Another important pathogen transmitted by fleas is the *R. felis*, a worldwide distributed rickettsia that causes the flea-borne spotted fever. This bacterium may be transmitted by several flea species but has the *C. felis felis* as its main vector (Angelakis et al. 2016). Just like in the murine typhus, opossums are suggested as potential reservoirs of *R. felis* (Boostrom et al. 2002; Brown and Macaluso 2016). For instance, studies performed on *D. virginiana* reported high seroprevalence for *R. felis* (i.e., 22%) as well as the presence of the bacterium DNA in tissue (e.g., spleen, liver, and kidney) and in *C. felis felis* fleas collected on them (Boostrom et al. 2002; Karpathy et al. 2009; Panti-May et al. 2015). However, the role played by these animals as reservoir of *R. felis* may vary according to the opossum species and further investigations are needed, since undetectable rickettsemia was reported in *D. aurita* in Brazil following experimental infection (Horta et al. 2010). Fleas are also known as vectors of the deadly bacteria *Y. pestis* (Pechous et al. 2016). Up to date, this bacterium has been detected through serological and bacterial culture in *D. albiventris* in Brazil (Almeida et al. 1987; Almeida et al. 1995). Indeed, considering the infestation of the main vector of this bacterium (i.e., *X. cheopis*) on *D. aurita* (Bezerra-Santos et al. 2020b), it is worth to further investigate the role of these animals as reservoir of this pathogen.

**Table 2:** Fleas reported on *Didelphis* species, occurrence on humans and associated zoonotic pathogens

Marsupial species	Flea species	Occurrence on humans	Associated pathogens	Reference
***Didelphis albiventris***	*Ctenocephalides felis felis*	Yes	*Rickettsia felis, Bartonella* spp., *Dipylidium caninum*	Horta et al. (2007); Kumsa et al. (2014); Youssefi and Rahimi (2014); Fontalvo et al. (2017); Silva et al. (2016); Abdullah et al. (2019); Urdapilleta et al. (2019)
	*Polygenis atopus*	No record	*Rickettsia felis*	Lareschi et al. (2006); Horta et al. (2007)
	*Tunga penetrans*	Yes	No record	Pampiglione et al. (2009); Danilo Saraiva et al. (2012)
***Didelphis aurita***	*Ctenocephalides felis felis*	Yes	*Rickettsia felis, Bartonella* spp., *Dipylidium caninum*	Horta et al. (2007); Youssefi and Rahimi (2014); Kumsa et al. (2014); Abdullah et al. (2019)
	*Adoratopsylla antiquorum*	No record	No record	Pinto et al. (2009)
	*Adoratopsylla intermedia copha*	No record	No record	Salvador et al. (2007); Pinto et al. (2009)
	*Leptopsylla segnis*	No record	*Rickettsia felis*	Salvador et al. (2007); Christou et al. (2010)
	*Polygenis atopus*	No record	*Rickettsia felis*	de Moraes et al. (2003); Horta et al. (2007)
	*Polygenis occidentalis*	No record	No record	Pinto et al. (2009)
	*Polygenis rimatus*	No record	No record	Urdapilleta et al. (2019)
	*Xenopsylla cheopis*	Yes	*Rickettsia typhi, Rickettsia felis, Yersinia pestis*	Brouqui et al. (2005); Salvador et al. (2007); Eremeeva et al. (2008); Christou et al. (2010); Hinnebusch et al. (2017)
***Didelphis marsupialis***	*Adoratopsylla intermedia copha*	No record	No record	Beaucournu et al. (1998)
	*Ctenocephalides felis felis*	Yes	*Rickettsia felis, Bartonella* spp., *Dipylidium caninum*	Beaucournu et al. (1998); Youssefi and Rahimi (2014); Kumsa et al. (2014); Abdullah et al. (2019)
	*Polygenis beebei*	No record	No record	Beaucournu et al. (1998)
	*Polygenis klagesi*	No record	No record	Beaucournu et al. (1998)
***Didelphis virginiana***	*Cediopsylla simplex*	No record	No record	Durden and Wilson (2016)
	*Ctenocephalides felis felis*	Yes	*Rickettsia felis, Bartonella* spp., *Dipylidium caninum*	Durden and Wilson (2016); Pung et al. (1994); Eremeeva et al. (2012); Youssefi and Rahimi (2014); Kumsa et al. (2014); Maina et al. (2016); Abdullah et al. (2019)
	*Ctenophthalmus pseudagyrtes*	No record	*Bartonella vinsonii*	Durden and Wilson (2016); Reeves et al. (2007)
	*Echidnophaga gallinaceae*	No record	*Rickettsia felis*; *Yersinia pestis*	Eremeeva et al. (2012); Jiang et al. (2013); Ehlers et al. (2020)
	*Orchopeas howardi*	No record	*Bartonella* sp.	Durden and Wilson (2016); Reeves et al. (2007)
	*Polygenis gwyni*	No record	*Bartonella* spp.	Pung et al. (1994); Abbot et al. (2007)
	*Pulex irritans*	Yes	*Yersinia pestis, Bartonella* spp.	Eremeeva et al. (2012); Ratovonjato et al. (2014); Dean et al. (2018); Fontalvo et al. (2017)
	*Pulex simulans*	No record	*Bartonella* spp.	Pung et al. (1994); Durden and Richardson (2013); Gabriel et al. (2009); López-Pérez et al. (2017)
	*Xenopsylla cheopis*	Yes	*Rickettsia typhi, Rickettsia felis, Yersinia pestis*	Brouqui et al. (2005); Eremeeva et al. (2008); Christou et al. (2010); Eremeeva et al. (2012); Hinnebusch et al. (2017)

### Phlebotomine sand flies and leishmaniasis

Opossums have been identified as a blood source for several hematophagous arthropods, including phlebotomine sand flies (e.g., *Lutzomyia longipalpis* and *Lutzomyia evansi*), which are accounted as vectors of pathogenic *Leishmania* spp. (Adler et al. 2003; Guimarães-e-Silva et al. 2017) such as *L. infantum*, the etiological agent of American visceral leishmaniasis (Spiegel et al. 2016; Mejía et al. 2018). Indeed, natural and experimental infection with species of zoonotic *Leishmania* (e.g., *L. infantum*, *L. brasiliensis*) in *Didelphis* spp. confirm their participation in the sylvatic and peridomestic cycles of these protozoa (Schallig et al. 2007; Carreira et al. 2012; Humberg et al. 2012; Silva et al. 2016). Natural infection by different *Leishmania* spp. has been reported in these marsupials in the New World (Table 3) (Santiago et al. 2007; Quintal et al. 2011; Carreira et al. 2012; Lima et al. 2013; Silva et al. 2016; Maia et al. 2018). For example, in urban and peri-urban areas of Southeastern Brazil, *D. albiventris* and *D. aurita* opossums displayed a high prevalence (i.e., 91.96%; *n* = 103/112) of *L. infantum* through PCR screening of DNA isolated from bone marrow, supporting their role as hosts of the causative agent of visceral leishmaniasis (Santiago et al. 2007). The role of marsupials in the epidemiology of this disease has been observed particularly in areas with peridomestic transmission, where phlebotomine sand flies may feed on opossums and eventually on other susceptible hosts, including people and domestic animals (e.g., dogs, cats), with opossums being suggested as a link in the transmission of visceral leishmaniasis to humans and dogs in rural and urban environments (Carranza-Tamayo et al. 2016). This has been further supported in an endemic area of Northeastern Brazil, where it was demonstrated that the months with higher opossum population density (rainy and colder) correlates with the peak of *Lu. longipalpis* population and with new cases of visceral leishmaniasis in humans (Sherlock 1996). In the same study *D. albiventris* capacity to infect *Lu. longipalpis* with *L. infantum* was confirmed through xenodiagnoses, in which 14% (*n* = 27/193) of the sand flies scored positive for this protozoan after feeding on a naturally infected opossum (Sherlock 1996). Data were confirmed through the experimental infection of *D. marsupialis* with *L. infantum*, which was able to infect *Lu. longipalpis* following blood feeding on this opossum species (Travi et al. 1998). In addition, though a small proportion (i.e., 2.6%; *n* = 8/312) of sand flies got infected after feeding on *D. marsupialis*, a high attraction rate for sand flies in trapped opossums was registered by the authors, strengthening their possible role as reservoirs for *L. infantum* (Travi et al. 1998). Finally, the detection of human and *D. albiventris* DNA in *Lu. longipalpis*, allowed to establish a link between these two hosts in an area of Brazil endemic for visceral and cutaneous leishmaniasis (Guimarães-e-Silva et al. 2017).

**Table 3:** Zoonotic helminths and protozoa reported in *Didelphis* species

Marsupial species	Endoparasites	Reference
***Didelphis albiventris***	**Helminths**	
	*Toxocara cati* (Nematoda)	Pinto et al. (2014)
	*Trichinella spiralis* (Nematoda)	Castaño Zubieta et al. (2014)
	*Schistosoma mansoni* (Trematoda)	Kawazoe et al. (1978)
	*Paragominus mexicanus* (Trematoda)	Blair et al. (1999)
	**Protozoa**	
	*Leishmania amazonensis*	Maia et al. (2018)
	*Leishmania braziliensis*	Silva et al. (2016)
	*Leishmania infantum*	Humberg et al. (2012)
	*Toxoplasma gondii*	Fornazari et al. (2011)
	*Trypanosoma cruzi*	Lima et al. (2012); Tenório et al. (2014); Drozino et al. (2019)
***Didelphis aurita***	**Helminths**	
	*Ancylostoma caninum* (Nematoda)	Bezerra-Santos et al. (2020a)
	*Schistosoma mansoni* (Trematoda)	Coelho et al. (1979)
	**Protozoa**	
	*Leishmania infantum*	Carreira et al. (2012)
	*Toxoplasma gondii*	Pena et al. (2011); Bezerra-Santos et al. (2020c)
	*Trypanosoma cruzi*	Teodoro et al. (2019)
***Didelphis marsupialis***	**Helminths**	
	*Schistosoma haematobium* (Trematoda)	Kuntz et al. (1971); Kuntz et al. (1975)
	*Paragonimus caliensis* (Trematoda)	Blair et al. (1999)
	*Paragominus mexicanus* (Trematoda)	Blair et al. (1999); López-Caballero et al. (2013)
	**Protozoa**	
	*Leishmania amazonensis*	Maia et al. (2018)
	*Leishmania guyanensis*	Maia et al. (2018)
	*Leishmania mexicana*	Maia et al. (2018)
	*Leishmania panamensis*	Maia et al. (2018)
	*Leishmania braziliensis*	Schallig et al. (2007)
	*Leishmania infantum*	Travi et al. (1994);
	*Toxoplasma gondii*	Yai et al. (2003)
	*Trypanosoma cruzi*	Galaviz-Silva et al. (2017)
***Didelphis virginiana***	**Helminths**	
	*Spirometra mansonoides* (Cestoda)	Corkum (1966)
	*Toxocara canis* (Nematoda)	Blumenthal and Kirkland (1976)
	*Trichinella spiralis* (Nematoda)	Leiby et al. (1988)
	*Angiostrongylus cantonensis* (Nematoda)	Kim et al. (2002); Dalton et al. (2017)
	*Angiostrongylus costaricensis* (Nematoda)	Miller et al. (2006)
	*Paragonimus kellicotti* (Trematoda)	Blair et al. (1999)
	*Paragominus mexicanus* (Trematoda)	Blair et al. (1999); López-Caballero et al. (2013)
	*Alaria marcianae* (Trematoda)	Shoop and Corkum (1981)
	**Protozoa**	
	*Toxoplasma gondii*	Torres-Castro et al. (2016)
	*Trypanosoma cruzi*	Parada-López et al. (2013); Cantillo-Barraza et al. (2015);Ruiz-Piña and Cruz-Reyes (2002)

### Triatomines and *Trypanosoma cruzi*

*Trypanosoma cruzi* is the etiological agent of Chagas disease, a neglected tropical illness mostly associated to poor housing conditions of the population in developing countries (Barbosa-Silva et al. 2019). Opossums play an important role in the zoonotic cycle of *T. cruzi* (Legey et al. 2003; Cantillo-Barraza et al. 2015; Jansen et al. 2015, 2017). This protozoan may be transmitted to humans via fecal matter of its insect vectors (i.e., triatomines) or by the consumption of contaminated food due to the presence of infected triatomines during the preparation of food items (e.g., açaí palm fruit, sugar cane, mango), ingestion of raw meat of infected animals, and through breast feeding (de Noya and González 2015; Santana et al. 2019). *Didelphis* spp. may be associated with both transmission routes in the epidemiological cycle of *T. cruzi* (Jansen et al. 2015, 2017). Indeed, the interaction of these opossums with triatomine insects (i.e., detection of opossums blood in triatomines, and infection of opossums after being exposed to infected triatomines), as well as the high prevalence of *T. cruzi* infecting the anal glands (optimal microenvironment for the development of infective metacyclic stages of this parasite) of these marsupials suggest their participation in the epidemiological cycle involving vector and oral transmission of *T. cruzi* (Steindel et al. 1988; Schweigmann et al. 1995; Urdaneta-Morales and Nironi 1996; Zecca et al. 2020). For example, bloodmeal analysis of *T. cruzi-*infected triatomine (*Triatoma gerstaeckeri*) collected in a dog shelter in South Texas, USA revealed the presence of canine, opossum, and human blood, suggesting the exchange of this protozoan among these hosts (i.e., opossums, dogs and humans) around urban dwellings, and the participation of opossums in the spreading of *T. cruzi* in endemic areas as these animals are known to circulate among different geographical sites (Zecca et al. 2020).

The presence of *T. cruzi* in opossum’s anal glands has been suggested as an oral transmission route since infective forms of this protozoan can be released in the environment, contaminate food, or directly, skin and mucosa of susceptible hosts (Steindel et al. 1988; Urdaneta-Morales and Nironi 1996). This has been demonstrated through the oral infection of mice with metacyclic forms obtained from the anal glands of *Didelphis* spp. (Steindel et al. 1988; Urdaneta-Morales and Nironi 1996), which led to the suggestion of the involvement of opossums in Chagas disease outbreaks via oral infection in areas where the presence of insect vectors was not detected (Steindel et al. 1988). Additionally, the use of these marsupials as food source for humans may also be implicated as a potential risk for the transmission of *T. cruzi* due to the consumption of undercooked opossum meat (Carvalho et al. 2020; Sangenis et al. 2015; Sangenis et al. 2016).

## Endoparasites associated with *Didelphis* spp.

### Helminths

Zoonotic helminths parasitizing opossums of the genus *Didelphis* have been reported throughout the American continent (Table 3), with some species being considered accidental parasitism (e.g., *Toxocara cati* in *D. albiventris*; Pinto et al. 2014) and others being recently identified with high prevalence rates (e.g., *Ancylostoma caninum* in *D. aurita*; Bezerra-Santos et al. 2020a). Among the helminth species harbored by opossums, *Trichinella spiralis*, *Angiostrongylus cantonensis*, *Angiostrongylus costaricensis, Paragonimus* spp., *Alaria marcianae*, and *Echinostoma* spp. (Table 3) present major public health importance due to the disease they cause in humans. *Trichinella spiralis* is a zoonotic nematode worldwide distributed and transmitted to humans by the ingestion of undercooked meat, especially pork (Diaz et al. 2020). The advances in hygiene in the pork industry has made the transmission of this parasite via commercial pork meat less prone to occur; therefore, the consumption of raw or undercooked meat of wildlife reservoirs (e.g., wild boar, bear, deer, moose, and walrus) is now the most frequent form of transmission (Diaz et al. 2020). In this perspective, the occurrence of *T. spiralis* in *Didelphis* spp. has a great epidemiological importance and should be considered for investigation, as these animals’ meat, usually from non-legalized game hunting, are consumed by humans in many regions of South America (Júnior et al. 2010; Barros and Azevedo 2014; de Oliveira Carneiro et al. 2019). Opossums have also been reported harboring *A. cantonensis* (Kim et al. 2002; Dalton et al. 2017), a zoonotic nematode recognized as a primary cause of eosinophilic meningitis in humans (Wang et al. 2008; Barratt et al. 2016). The biological cycle of this nematode involves rats as definitive hosts, snails, and slugs as intermediate hosts, and crustaceans, predacious land planarians, frogs, and lizards as paratenic hosts (Mendoza-Roldan et al. 2020b), as well as the transmission of infective L3 from an infested snail to another (i.e., intermediesis; Modrý et al. 2020). Humans become infected by ingesting third-stage larvae (L3) present in intermediate or in paratenic hosts, as well as contaminated vegetables (Wang et al. 2008). Up to date, this nematode has been detected only in *D. virginiana*; infected individuals present weakness, ataxia and neurological abnormalities such as circling due to the presence of adult worms in the brain tissue (Kim et al. 2002; Dalton et al. 2017). *Angiostrongylus costaricensis* has also been reported in a *D. virginiana* opossum causing localized peritonitis with adhesions from omentum and presence of adult worms (24 females and 12 males) in the mesenteric arteries (Miller et al. 2006). Similarly, to *A. cantonensis*, the life cycle of *A. costaricensis* also involves rats as definitive hosts, and snails and slugs as intermediate hosts, with human and other mammal infections happening through the ingestion of intermediate hosts containing L3, as well as of contaminated vegetables (Miller et al. 2006). The role of opossums in the biological life cycle of *A. cantonensis* and *A. costaricensis* is still unknown and deserves further studies.

Opossums have also been reported harboring zoonotic trematodes, such as lung flukes of the genus *Paragominus*, with the species *Paragonimus caliensis*, *Paragonimus kellicotti*, and *Paragominus mexicanus* being described in these marsupials in the Americas (Table 3) (Blair et al. 1999; López-Caballero et al. 2013). These parasites have a life cycle comprised by two intermediate hosts (aquatic snails and crustaceans), and several mammal species as definitive hosts (Blair et al. 1999). In the Americas, opossums are among the definitive hosts for *P. caliensis*, *P. kellicotti*, and *P. mexicanus*, playing, along with intermediate hosts, a key role in the maintenance of these parasites in nature (Blair et al. 1999). Another parasite species associated with opossums is the trematode *A. marcianae*, reported on *D. virginiana* in Louisiana, USA (Shoop and Corkum 1981). The life cycle of this trematode involves two intermediate hosts (i.e., snails and amphibians) and a definitive host (i.e., canids, felids, or mustelids), with opossums being considered paratenic hosts (Möhl et al. 2009). Indeed, the consumption, or even manipulation (e.g., skinning, evisceration) of paratenic hosts have been implicated as a source of *Alaria* spp. infection to humans (Shoop and Corkum 1981; Möhl et al. 2009), highlighting the risks of the consumption of opossums by people. Finally, *Gnathostoma* spp. and *Echinostoma* spp. have been associated with opossums; however, although several species of these parasites are zoonotic, the ones reported (i.e., *Gnathostoma turgidum* and *Echinostoma trivolvis*) on these marsupials have not been proven to affect humans or domestic animals (Alden 1995; Maldonado and Lanfredi 2009; Torres-Montoya et al. 2018).

### Protozoa not vectored by arthropods

Parasitic protozoa are particularly important due to their wide host range, which include humans, domestic and wild animals. Additionally, many of these parasites are responsible for important economic losses related to farm animals, and for causing disease in humans (Sahinduran 2012; Kaltungo and Musa 2013). In opossums, most studies on zoonotic protozoa infecting these marsupials described blood and gastrointestinal species of public health importance, such as *Trypanosoma*, *Toxoplasma*, *Leishmania* and *Cryptosporidium* spp. (Table 3). In this section we will focus on the gastrointestinal protozoa of public health concern (for vector-borne protozoa see sections 3.3 and 3.4).

Several studies have speculated about the role of opossums as reservoirs of *T. gondii* (Yai et al. 2003; Fornazari et al. 2011; Suzán and Ceballos 2005; Gennari et al. 2015). In fact, this protozoan has been molecularly and serologically detected in *Didelphis* spp. For example, *T. gondii* in these animals have been reported with seroprevalence of 37.3% (*n* = 148/396 by indirect immunofluorescent antibody test – IFAT) in *D. marsupialis*; 5.55% (*n* = 4/72 by modified agglutination test - MAT) in *D. albiventris*; 10.34% (*n* = 3/29 by complement fixation test) in *D. virginiana*, and 12.5% (*n* = 5/40 by MAT) in *D. aurita* (Yai et al. 2003; Fornazari et al. 2011; Suzán and Ceballos 2005; Gennari et al. 2015). Additionally, DNA of this protozoan has been isolated from heart and brain tissues of *D. aurita* and *D. virginiana*, respectively (Pena et al. 2011; Torres-Castro et al. 2016). Finally, these animals are considered a food source in some regions, and the consumption of undercooked meat of these animals have been implicated as a potential risk for the transmission of *T. gondii* (Alvarado-Esquivel et al. 2016). However, their role in the transmission cycle of *T. gondii* remains unclear.

Other gastrointestinal protozoa (e.g., *Cryptosporidium* spp., and *Giardia* spp.) may be spread through contaminated feces of opossums in food and water (Oates et al. 2012). Studies on both the protozoa above affecting *Didelphis* spp. are scant, and most of them rely on the identification to the genus level (Zanette et al. 2008; Oates et al. 2012), which makes difficult the assessment of their zoonotic potential. Indeed, up to date only one experimental study performed in *D. virginiana* has demonstrated the infection of the zoonotic protozoan *Cryptosporidium parvum* in these animals, with four out of seven infected nursing opossums presenting mild clinical signs, such as diarrhea (Lindsay et al. 1988); however, factors such as age (youngsters, which may not present a completely developed immune system), high doses of *C. parvum* oocysts inoculated (5 x 100^6^), and the lack of detection of natural infection of *C. parvum* in these marsupials make it unclear whether they are involved in the transmission of this parasite.

## Opossums as a source of parasitic infections to domestic animals

The relationship between opossum and domestic animals has been recorded in several regions where these marsupials occur, with the direct and indirect contact among them, implicating in the transmission of parasites of animal health concern. For example, a high prevalence of *A. caninum* was detected in *D. aurita* in southeastern Brazil (Bezerra-Santos et al. 2020a), although the relative importance of opossums in transmitting this parasite to dogs and other susceptible hosts is unclear. Opossums have also been involved in the transmission of parasites of concern to farm animals. This is the case of *D. virginiana*, the definitive host of *Sarcocystis neurona*, a protozoan parasite known to cause severe and fatal neurologic disease in horses (Rossano et al. 2003). The above-mentioned examples are among the few studies investigating the importance of opossums in the epidemiology of parasitic diseases to domestic animals. Indeed, considering their circulation and contact with pets in urban environments, and with livestock in rural settings, these marsupials could be playing an underestimated role in the epidemiology of parasitic diseases affecting domestic animals and livestock.

## Conclusions

The ecological role of native species is essential for the equilibrium of an ecosystem. Anthropogenic activities and invasion of natural habitats of endemic wild species have negative consequences not only for the wildlife, but also for the health of humans and domestic animals, as many wildlife species are involved in the transmission of different zoonotic pathogens. Opossums are good examples of such animals, due to the direct (e.g., hunting, manipulation and consumption of their meat, illegal trade in local markets) and/or indirect contact (e.g., ectoparasites, contaminated food and water) of people and domestic animals with these marsupials. This situation brings important risks from a “One Health” point of view, as infectious agents may cause disease in some species (e.g., *R. rickettsii* in humans), but use other hosts only as reservoirs (e.g., *R. rickettsii* in opossums). Despite presenting substantial importance in a One Health context, knowledge on infectious agents of public and veterinary importance associated with opossums are still scant, advocating further research on the role these animals play in the epidemiology of such pathogens. In addition, the education of the population about the risks brought by the direct contact with such animals is pivotal to reduce the risks of sharing pathogens among marsupials, domestic animals, and humans.
